# Economic and Social Impacts of COVID-19 on Animal Welfare and Dairy Husbandry in Central Punjab, Pakistan

**DOI:** 10.3389/fvets.2020.589971

**Published:** 2020-10-23

**Authors:** Sabir Hussain, Abrar Hussain, Jeffery Ho, Olivier A. E. Sparagano, Ubaid-ur-Rehman Zia

**Affiliations:** ^1^Department of Epidemiology and Public Health, The University of Veterinary and Animal Sciences, Lahore, Pakistan; ^2^Department of Infectious Diseases and Public Health, Jockey Club College of Veterinary Medicine and Life Sciences, City University of Hong Kong, Hong Kong, China

**Keywords:** animal health, economic losses, milk production reduction, COVID-19, animal feeding shortage

## Abstract

Studies on the impact of the COVID-19 pandemic on animal welfare and dairy husbandry in low-income countries are limited. We conducted a survey between February and June 2020 to evaluate the economic impact and animal health problems upon the pandemic. Participants were lead veterinarians from 14 dairy farms with herd size between 100 and 500 in Lahore. These farms were major suppliers of milk and dairy products to central Punjab, Pakistan. During the pandemic, 10 of the 14 dairy farms relied on feed mill concentrates to provide feeds to their herds. Half of the farms reported feed shortage due to lockdowns. Six (43%) dairy farms have witnessed a 7.5% shortage of dry feed intake. In seven (50%) farms, the body condition score decreased by 0.24 point. The body score reduction was significantly associated with depleted feed intake (*P* = 0.005). The veterinarians of 10 (71%) farms failed to gain access to essential veterinary medications, hampering the treatment of sick animals. Due to feed shortage and drug unavailability, daily milk production reduced by two litters per cow in the herd of five (35%) farms. The reduced feed intake was significantly associated with the decrease in milk production (*P* = 0.003), while numerous downstream milk-processing facilities were out of service during the pandemic, significantly reducing the profit of six (43%) dairy farms. Finally, our study showed that the dairy farming industry and animal welfare were critically affected by three aspects: feed shortage, inaccessibility to essential veterinary drugs, and a reduced consumer demand for dairy products.

## Introduction

The most recent strain of coronavirus COVID-19 belongs to the SARS-CoV-2 group. This COVID-19 pandemic started from the Wuhan, Hubei Province, China (1, 2). This pandemic continued to spread relentlessly (3). COVID-19 was declared as a Public Health International Emergency by the World Health Organization on January 30, 2020 (4). The number of confirmed coronavirus cases was around 27 million cases and confirmed deaths around 0.9 million globally (as of September 7, 2020) (5). This pandemic is still affecting many industries, including agriculture and the food system and damaging the livelihood of many people (1, 6, 7). COVID-19 has affected food security and the poorest nations are under an increasing risk of hunger and food chain disruption. All over the world, chronic hunger affected 820 million people, and acute severe insecurity was also reported for 113 million people (8). The well-being of the farmers and the supply of safe and healthy foods should be ensured in such disaster condition through proper strategies (9). The Organization for Economic Cooperation and Development (OECD) has predicted that economic growth will go down from 2.9 to 2.4% in 2020; it may go down to 1.5% if the pandemic is prolonged (10, 11). It is estimated that 60% of the world population depends on agriculture for survival, and COVID-19 is affecting different agriculture sectors including crop production and livestock (9, 12). The economic growth of dairy farms has been hit hard by COVID-19 all over the world. Milk processing units are closed, which created a gap between demand and supply chains during COVID-19. This situation forced the dairy farmers to dump 4 million gallons of milk in the USA in the first week of April (13). In Nepal, COVID-19 has damaged ~17 million US dollars of dairy products, and dairy products worth 42 million US Dollars are at risk of deterioration (14). A similar study was conducted in Canada and showed the closure of many retailing shops. Fresh milk demand went down and dairy farmers could not store the milk for many days. On the other hand, processing units are also at their full capacity, so farmers had to dump their stored milk to make room for fresh milk (15). In Bangladesh, dairy farmers are also unable to sell their milk and are facing poor economic conditions due to lockdowns. Every day Bangladesh is incurring almost 67 million US dollars of losses due to the wastage of 15 million liters of milk. Furthermore, farmers were forced to sell their milk at a low rate circa 0.14 US dollar per litter, which was almost 0.6 US dollar less as compared to normal price (16). Pakistan is the third largest milk-producing country in the world and has 47.8 million cattle with an annual milk production circa 57.3 billion liters. Almost 23.2 billion liters are sold untreated on markets through a supply chain that lacks suitable storage and proper temperature control conditions. The remainder is mostly used in the preparation of traditional products such as ghee, butter, sweets, and yogurts (17).

This pandemic caused acute global socioeconomic problems such as fear of supply shortage, which resulted in panic buying (5, 18); 41% of exports have been affected all over the world by this pandemic (19). According to the International Labor Organization (ILO), 2.7 billion workers are affected due to partial or complete lockdown during this pandemic as this RNA virus can be transmitted from person to person through airborne particles and droplets (10, 20). COVID-19 cases in 115 meat processing facilities were reported among US workers by 19 states (21). The American Veterinary Medical Association (AVMA) has raised concerns due to the shortage of veterinary drugs from animal pharmaceutics due to panic buying. Such shortage may affect animal health and welfare (19). The main objective of our study was to analyze how the COVID-19 and its resulting lockdowns have been affecting the animal welfare and economic status of dairy farmers in central Punjab, Pakistan.

## Materials and Methods

The standardized questionnaire was generated and designed to assess the economic and social impacts of COVID-19 on animal welfare and dairy husbandry in Central Punjab, Pakistan. Only those dairy farms having an animal range between 100 and 500 and located within the boundaries of Central Punjab, Pakistan, were selected. All 14 farms had a mixed herd of Holstein Friesian and local breeds with double bucket milk line systems. The dairy farms included in our study mainly depended on feed-mill for concentrate feeds instead of grains. They used, before the pandemic, to rely on corn silage, alfalfa (*Medicago sativa*), and barseem (*Trifolium alexandrinum*) as roughages according to the seasonal availability. The 14 farm managers/veterinarians were working on these progressive dairy farms. Quantitative variables like drop in feed intake and drop in milk production were estimated from the previous record of weighing the feed before being offered to animals and following the recording of average milk production on each farm. The drop in body condition score (BCS) was estimated by one of the co-authors (9). The survey was conducted by visiting each dairy farm and adopting strict hygienic measures and protocols as per the government's recommendations. All the questionnaires were filled in by each veterinarian working on these dairy farms and talking to the farm owner. The data were entered and analyzed using the open source version SPSS-25.0. All the considered factors were analyzed using chi-square tests, while positive and negative correlations were done by using the bivariate correlation test.

## Results

In this study, dairy farms were economically underperforming, while animal welfare issues were observed. We found that ten farms (71%) were purchasing feeds from external companies rather than using their own feed mill on the farm. Feed shortage hit hard seven farmers with up to 50% of their supply becoming unavailable. During this pandemic, eight (57%) changed their feeding plans. Four farmers (28%) changed the commercial brand of feed due to availability changes, which caused a drop in feed-intake of circa 7.5%. Animal welfare was affected by this pandemic through feed shortage and drug unavailability causing an average drop of 0.24 point in the body condition score (BCS 1-5) for seven (50%) farms. Furthermore, six (43%) veterinarians said that cows' welfare was affected during this crisis. Our study found out that three (21%) veterinarians also reported a negative effect on calf health due to supply issues of calf-starter feeds during this period. However, the majority of the farms in this study did not have any calves during our visit so we could not evidence such claim. Further economic losses were also due to a decrease in milk production, which was observed on five (36%) dairy farms. Furthermore, due to the closure of milk processing units, during lockdowns, farmers have been facing problems through their milk supply chains at six (43%) out of 14 farmers. Finally, farmers were in poorer economic situations due to several negative drivers mentioned above (Table 1).

**Table 1:** Frequencies of responses at selected dairy farms.

**Question**	**Response**	**Frequency**	**Percentage**
Shortage of feed observed?	Yes	7	50
	No	7	50
How much shortage of feed observed?	Up to 50%	7	50
	No Loss	7	50
Did you change your feeding plan?	Yes	8	57
	No	6	43
Did you change the feed brand?	Yes	4	29
	No	10	71
Did you change your feeding plan? or same crude protein	Yes	9	64
	No	5	36
Any drop in feed intake?	Yes	6	43
	No	8	57
How much drop in feed intake %	7.5%	6	43
Drop in body score	Yes	7	50
	No	7	50
How much drop in body score (average)?	0.24	7	50
Was any drop in milk production?	Yes	5	36
	No	9	64
How much drop in milk litter per cow per day?	2	5	36
Any decrease in availability of calf-starter feed?	Yes	1	7
	No	13	93
Was the calf health affected?	Yes	3	21
	No	11	79
Problem in milk supply?	Yes	6	43
	No	8	57
Any economic losses sustained?	Yes	7	50
	No	7	50
Problem in drug availability?	Yes	10	71
	No	4	29
Was your cows welfare affected?	Yes	6	43
	No	8	57

The chi-square test was applied to check the association between two variables i.e., changes in feed brand and drop-in feed intake. Results [χ^2^(1) = 7.47, *P* = 0.015] showed clearly that there was an association, which was statistically significant and caused a drop in feed intake by changing the feed brand. Such drop of feed intake also affected the animal health, and to analyze this effect, we applied the chi-square test and found the following results: χ^2^(1) = 10.50, *P* = 0.005 showing a strong association of the drop-in body score with the decrease in feed intake. It also contributed to economic losses by decreasing milk production. A chi-square test showed a strong correlation between such parameters: χ^2^(1) = 10.37, *p* = 0.003 showing a statistically significant association between the drop in feed intake and milk production. Similarly, the association of drop in milk production with drug unavailability showed the following results χ^2^(1) = 3.10, *P* = 0.221 highlighting a lack of association between them. Therefore, drug unavailability is a less contributing factor compared to a drop in feed intake for milk production reduction (Table 2).

**Table 2:** Chi-Square analysis of associated risk factors.

**Question**	**χ^**2**^**	***P*-value**
**Responses with comparison of drop in feed intake**		
Feed brand change	7.47	0.015
Drop in body condition score	10.50	0.002
Drop in milk production	10.37	0.003

For the quantitative variable analysis, we used a bivariate correlation test i.e., to check the correlation between the drop in feed intake and the drop in BCS. We found: *r*(1) = 0.70, *P* = 0.006, which is showing a strong positive correlation between them during this pandemic period. Milk reduction was always affected by the feed intake. Our data, using a bivariate correlation test, showed: *r*(1) = 0.76, *p* = 0.002, which is a showing a strong significant correlation. Results from the quantitative data analysis highlighted that both animal health and animal products, which are the backbone of the dairy sector, were highly affected by a drop in feed intake. COVID-19 is a key driver causing feed shortage, which leads to changing feed plans as well as the concentrate feed brands ultimately influencing negatively the feed intake (Table 3).

**Table 3:** Correlation analysis of quantitative variables.

**Question**	***r***	***P*-value**
**Responses with comparison in reduction in feed intake**		
Drop in body condition score	0.70	0.006
Drop in milk production	0.76	0.002

## Discussion

The global pandemic of COVID-19 affected the respective national gross domestic products (GDPs) ranging from 0.1 to 0.4%. However, this pandemic decreased the tourism arrival in many developing Asian economies by 50 to 90% compared to the previous year (9, 22). In India, during lockdown, the agricultural sector and many supply chains (distribution, transport hurdles, marketing and processing) were greatly affected (23). Closure of restaurants and transport restrictions reduced the demand of fresh dairy products, affecting producers and suppliers (19). In Pakistan, the real growth in GDP is expected to decrease by 3% due to this pandemic and along with other industries such as agriculture and livestock farming, which are also under threat of severe economic losses (24).

This pandemic played a role in increasing damage to animal welfare (25, 26) Our study found many negative impacts from COVID-19 on the animal welfare and production capacity of the dairy sector in Pakistan. The major issue was the limitation in feed availability for the dairy farms due to the closure of feed mills during the lockdown and the lack of commercial feed availability, which resulted major economic losses. According to the Ethiopian Country Commercial Guide (2020), milk demand decreased due to the closure of restaurants alongside consumers decreasing their demand of milk and dairy products by considering these products as a possible source of transmission of the coronavirus (27). However, according to the FAO and WHO guidelines, there is no evidence that these products acted as a source of transmission of the disease (28). Due to the perishable nature of dairy products, milk producers showed higher losses than supermarkets (13). Farmers had restricted options to supply their milk as a fresh product. As COVID-19 has driven a rapid and radical transformation from face-to-face care to telehealth in primary care practices, to ensure the animal welfare, telemedicine services are needed in this critical period (29). Novel coronavirus is not limited to humans, but recently, cats, dogs, lions, and tigers were diagnosed positive, but it had not been detected in livestock (30). Although the coronavirus can cross the interspecies barrier, it is too early confirm the role of intermediate hosts such as turtles, snakes, pangolins, and other wild animals in the origin of SARS-CoV-2 (31). There is dire need for strengthening of research, surveillance, and monitoring of animals for SARS-CoV-2 through the One Health approach. Veterinarians working on the farms in our study also observed problems regarding animal welfare such as the lack of drug availability for treating sick animals during this pandemic. There was also shortage of veterinary diagnostic services during the lockdown, and most diagnostic laboratories were focusing on COVID-19 leading to limited veterinary diseases diagnosis. Farmers and veterinarians should be involved in the current situation and the strategic future of the sustainable agriculture sector. During this disastrous period, it is the responsibility of the media (including social media) and veterinary professionals to spread awareness among the general public to avoid the condition to worsen and spread further (32, 33).

We concluded that there was a significant negative impact of COVID-19 on the surveyed farms on animal health, milk production, and animal welfare. Such issues were due to the closing of feed mills and transport hurdles, which created barriers in the milk supply chain. Dairy farmers also faced economic losses due to a decrease in milk production and limitations in selling milk. As milk production is the basic source of income for these dairy farmers, such pandemic greatly affected their livelihood as for those involved in hospitality industry, food and feed transportation, and the food chain. If the government does not provide proper support to the dairy sector at this time, then not only the dairy sector but other connected businesses will go down, and long-lasting consequences will remain beyond this pandemic.

## Data Availability Statement

The raw data supporting the conclusions of this article will be made available by the authors, without undue reservation.

## Ethics Statement

This survey-based study was conducted in a manner consistent with Animal Welfare Acts and did not require any ethics committee approval.

## Author Contributions

SH, AH, and U-u-RZ conceived the study, conducted the questionnaire and data entry, performed the statistical analysis, drafted the manuscript, and revised the manuscript. JH and OS provided intellectual input and critically revised the manuscript. All authors read and approved the final manuscript.

## Conflict of Interest

The authors declare that the research was conducted in the absence of any commercial or financial relationships that could be construed as a potential conflict of interest.
